# Impact of khat (*Catha edulis*) and oral contraceptive use on telomerase levels and tumor suppressor genes p53 and p21 in normal subjects and breast cancer patients

**DOI:** 10.1038/s41598-024-67355-5

**Published:** 2024-07-16

**Authors:** Fairooz Atroosh, Molham AL-Habori, Ekram Al-Eryani, Riyadh Saif-Ali

**Affiliations:** Department of Biochemistry and Molecular Biology, Faculty of Medicine and Health Sciences, Sana’a University, Sana’a, Republic of Yemen

**Keywords:** Khat, *Catha edulis*, Oral contraceptives, Breast cancer, Telomerase, Tumor suppresser gene, P53, P21, Biomarkers, Oncology, Risk factors

## Abstract

This study aimed to evaluate the effects of oral contraceptive (OC) use, khat chewing, and their combined effect on telomerase level and tumor suppressor genes, p53 and p21 in breast cancer (BC) patients and normal volunteers. 140 Yemeni women aged 25–40 years old enrolled, 60 newly diagnosed pretreated BC patients, and 80 control subjects. Venous blood (5 ml) was collected and the results showed BC patients to have significantly raised levels of telomerase, p53, and p21 compared to the control group. The use of OCs significantly raised telomerase in control group with no effect in BC patients; whereas p53 and p21 were significantly increased in BC patients. On the other hand, khat chewing significantly increased p53 in controls and BC patients, whereas p21 was significantly raised in BC patients. The combined use of OCs and khat chewing significantly increased telomerase and p53 in control group, and significantly increased p53 and p21 in BC patients. Telomerase was shown to be a risk factor (OR 4.4) for BC, and the use of OCs was a high-risk factor for increasing telomerase (OR 27.8) in normal subjects. In contrast, khat chewing was shown to be protective (OR 0.142), and the combined use of OCs and khat chewing decreased the risk factor of telomerase from OR 27.8 to 2.1.

## Introduction

Breast cancer (BC) is the most commonly diagnosed cancer and the leading cause of cancer deaths in women, with an estimated 2.3 million new BC cases in 2020^1,2^, and is expected to reach 3 million by 2040^3^. The incidence is highest in developed countries such as Australia, New Zealand, Western and Northern Europe; and lowest in developing countries such as Central America, Eastern and Middle Africa^1^. Breast cancer is a heterogeneous disease and has significant variability according to ethnicity and race with respect to incidence, clinical characteristics, and prognosis^4,5^. The main risk factors for BC are early menarche before the age of 12 years, late menopause above 55 years of age, nulliparity, miscarriages before the first full-term pregnancy, late age at first birth, shorter breastfeeding periods, use of menopausal hormone-replacement therapy or oral contraceptives (OCs), and family history of breast cancer^6,7^. Breast cancer is also related to environmental factors that include high socio-cultural levels, smoking, obesity, and low physical activity^8,9^. Despite major advancements in its early diagnosis and treatment, conventional therapeutic strategies have shown limitations in effectively treating BC. However, the advent of antibody–drug conjugates, a new class of drug designed to specifically deliver high-potent chemotherapeutic agents directly to cancer cells, has made a breakthrough in the treatment of BC patients^10–12^.

Oral contraceptives, which contain both levonorgestrel (a progestin) and ethinyl estradiol (an estrogen), adjust normal reproductive function by mimicking the action of estrogen. The role of estrogens in BC development is long-established by the reduction of BC risk among women treated with selective estrogen receptor modulators or by inhibiting the production of estrogens with aromatase inhibitors^13^. Several meta-analysis studies concluded that the use of OCs significantly increased the risk of BC when compared with never-users, regardless of contraceptive pill type^5,14–17^. The risk of BC was higher among women who currently or recently used OCs and this risk increased with longer durations of use^18–20^ or before the first full-term pregnancy^21^, and was more pronounced in obese women. Other studies showed that the use of OCs was associated with a transiently increased risk of BC^4^ and that the duration of its use was not associated with increased BC risk^22^. In contrast, other studies reported no such association between the use of OCs and the risk of BC^23–26^.

*Catha edulis* (khat) is an evergreen shrub belonging to the family Celastraceae. The habit of khat chewing has prevailed for centuries among populations in the Horn of Africa and the Arabian Peninsula, including Yemen. Although khat chewing is known to cause health issues in certain individuals, it is estimated that 20 million people worldwide are regularly chewing khat^27^. Khat has been a part of Yemeni culture for a long time and is used in virtually every social occasion. Nearly 90% of adult males and more than 50% of females chew khat for 3–4 h daily. The common adverse effects of *Catha edulis* are wide and variable^28,29^. Khat was reported to induce apoptotic cell death, mitochondria damage, reduced cell proliferation, and morphological features of autophagy in various leukemic cells^30–32^ and normal human keratinocytes and fibroblasts^33^. khat also induces the tumor suppressor and cellular stress sensor p53 expression in normal human oral keratinocytes and oral fibroblasts^34^, human ovarian adenocarcinoma SKOV3 cell line^35^, and in human liver cell line L02^36^.

Telomerase is a ribonucleoprotein enzyme that specifically maintains telomere integrity and restores its sequences lost during replication^37^. Telomerase is activated in most immortal cell lines in culture and most malignant tumors^38^ and is detected in 85–90% of human cancers including BC^39–41^. It also is increased in preinvasive lesions of the breast, such as ductal carcinoma in situ, suggesting that telomerase activity is activated early in breast carcinogenesis^42–44^. Several studies reported the involvement of p53 in the regulation of telomerase activity in mammary epithelial cells and BC^45–47^ via the down-regulation of hTERT transcription^46^. p53 is a cellular stress sensor protein that initiates cell cycle arrest and apoptosis in response to DNA damage and oxidative stress^48^. The p53 protein induces apoptosis via transactivation of p21, which inhibits cell cycle progression by blocking the kinase activity of cyclin-dependent kinase (CDKs), leading to cell cycle arrest^49^. p21 is a downstream target of the p53 protein and the p53-p21 axis is an established central pathway for cell cycle inhibition and tumor suppression^50^.

This study aimed to examine the effects of OCs use, khat chewing, and the combined effect of OCs and khat chewing on the levels of telomerase enzyme and the tumor suppressor genes, p53 and p21 in both BC patients and normal volunteers.

## Subjects and methods

### Study design, subjects and data collection

This case-control study involved 140 Yemeni women aged 25–50 years old; 60 breast cancer patients and 80 control subjects. Breast cancer patients were selected based on referral by an oncologist after confirmation by ultrasound examination, mammography, and histopathology et al.-Kuwait Hospital and Al-Hayat Center for Breast Cancer Diagnosis. These patients were newly diagnosed pretreated cases of BC grade I, or II. All participating women in both groups had regular menstrual cycles, and pregnant women were excluded from both groups. The studied groups were subdivided according to whether the individual participants were: (1) using contraceptive pills [microgynon Fe-contain—levonorgestrel (synthesized progesterone) 0.15 mg and Ethinylestradiol 0.03 mg] daily for ≥ 1 year; (2) chewing khat 3 days a week for ≥ 3 years; (3) using contraceptive pills daily for ≥ 1 year and chewing khat 3 days a week for ≥ 3 years. The study protocol was approved by the institutional review board (IRB) of the faculty of medicine and health sciences, Sana’a University, and all methods were performed in accordance with the relevant guidelines and regulations. Informed consent form was obtained from all participants after explaining the purpose and nature of the study.

Data was collected for cases and control subjects by in-person interview using a questionnaire that includes sociodemographic, reproductive history, and family history of cancers. The subjects’ height and weight were measured and BMI, defined as weight (kg)/height squared (m^2^), was calculated.

### Blood collection and biochemical analysis

Venous blood (5 ml) was collected from each woman into plain tubes for biochemical assay. The serum from each sample was separated within 30 min, aliquoted into three Eppendorf tubes, and immediately kept at − 20 °C for biochemical analysis. Enzyme-linked immunoassay (ELISA) kits were used to measure telomerase reverse transcriptase (TERT), tumor protein p53 (p53), and antioncogene p21 protein (Blue Gene, China).

### Statistical analysis

The statistical analyses were performed on social package of social sciences (SPSS) version 11.5 (SPSS Inc, Chicago, IL, USA). *T*-test was used to compare two groups and ANOVA was used for comparing more than two groups. Logistic regression was used to assess the risk factors for changes in telomerase in control subjects. The accepted level of significance was set below 0.05 (*P* < 0.05).

### Ethics approval and consent to participate

The study protocol was approved by the Institutional Ethical Committee, Faculty of Medicine and Health Sciences, Sana`a University. The study was in compliance with the Declaration of Helsinki for clinical research. All the recruited participants provided written informed consent before participating in the study.

## Results

Table 1 shows the characteristics of the study population. The mean age of BC patients was non-significantly (*P* = 0.163) lower as compared to the control group. Body mass index (BMI) was significantly (*P* = 0.001) lower in BC patients by 9.4% as compared to the control group. In contrast, smoking was significantly (*P* = 0.007) higher in BC patients with respect to the control group by two-fold. Family history of BC was significantly (*P* = 0.001) higher among BC patients by 5.3-fold than the control group, all of whom were first-degree relatives. The levels of telomerase, p53, and p21 in BC patients were significantly (*P* = 2.60 × 10^−6^, *P* = 5.10 × 12^−8^, p = 1.5 × 10^−7^) higher by 16.3%, 42.3%, and 49.5% respectively as compared to the control group.

**Table 1:** Characteristics of the study population.

	Control (n = 80)	Breast cancer patients (n = 60)	*P* value
Socio-demographic character			
Age (years)	51.47 ± 11.86	47.68 ± 10.81	0.163
BMI (kg/m^2^)	24.14 ± 4.72	21.87 ± 4.06	0.001
Smoking (%)	17.4	35	0.007
Menstrual and reproductive characters			
Menarche age (years)	13.15 ± 0.79	13.0 ± 1.2	0.444
Menopause (%)	61.6	55.0	0.378
Menopausal age (years)	49. 54 ± 4.1	48.08 ± 3.9	0.069
No. of full-term pregnancy (years)	6.81	5.73	0.025
No. of miscarriage (years)	1.31	1.34	0.894
Family history of breast cancer			
First-degree relative (%)	1.25	6.6	0.001
Biochemical analysis			
Telomerase (ng/mL)	3.31 ± 0.63	3.85 ± 0.66	**2.6** × **10**^**−6**^
p53 (pg/mL)	111.00 ± 44.60	158.00 ± 51.40	**5.12** × **10**^**−8**^
p21 (ng/mL)	2.99 ± 0.66	4.47 ± 2.28	**1.5** × **10**^**−7**^

Data presented as mean ± SD.

Significant values are given in bold.

The levels of telomerase were significantly (*P* = 6.3 × 10^−9^) higher in the OC users of the control by 27.2% as compared to their corresponding non-OC users, with no significant difference in p53 and p21 levels (Table 2). In contrast, both p53 and p21 were significantly (*P* = 0.044, *P* = 2.8 × 10^−6^) higher in the OC users of the BC patients by 17.2% and 51.6% as compared to their respective non-OC users, with no significant difference in telomerase level. Similarly, p53 and p21 were significantly (*P* = 1.1 × 10^−5^, *P* = 2.2 × 10^−9^) higher in the OC users of the BC group by 44.1% and 71.3% with respect to the OC users of the control group; with the telomerase being non-significantly different.

**Table 2:** Effect of oral contraceptives on telomerase, p53 and p21 levels in breast cancer patients and control group.

	Control		*P* value	Breast cancer patients		*P* value
	No OCs (n = 40)	With OCs (n = 40)		No OCs (n = 30)	With OCs (n = 30)	
Telomerase (ng/mL)	2.91 ± 0.52	3.70 ± 0.48	**6.3** × **10**^**−9**^	3.89 ± 0.63	3.80 ± 0.70	0.563
p53 (pg/mL)	103.00 ± 42.40	118.00 ± 45.90	0.157	145.00 ± 56.20	170.00 ± 46.10*	**0.044**
p21 (ng/mL)	2.84 ± 0.56	3.14 ± 0.61	0.361	3.55 ± 1.33	5.38 ± 2.67*	**2.8** × **10**^**−6**^

*Significant on comparing contraceptive pill users between groups (breast cancer vs. control) for p53 (*P* = 1.1 × 10^−5^), p21 (*P* = 2.2 × 10^−9^).

Significant values are given in bold.

The levels of p53 were significantly (*P* = 0.0003) higher in the khat chewers of the control group by 39.6% as compared to their corresponding non-khat chewer group, with no significant difference in telomerase and p21 (Table 3). However, both p53 and p21 were significantly (*P* = 0.001, *P* = 4.9 × 10^−5^) higher in the khat chewers of the BC patients by 27.3% and 43.6% as compared to their corresponding non-khat chewers BC patients, with no significant difference in telomerase level. However, telomerase, p53, and p21 were significantly (*P* = 0.001, *P* = 1.8 × 10^−5^, *P* = 2.2 × 10^−8^) higher in khat chewers of BC group than khat chewers of the control group by 16%, 37.2%, and 67.3% respectively.

**Table 3:** Effect of khat chewing on telomerase, p53 and p21 levels in breast cancer patients and control group.

	Control		*P* value	Breast cancer patients		*P* value
	No khat (n = 40)	Khat chewers (n = 40)		No khat (n = 30)	Khat chewers (n = 30)	
Telomerase (ng/mL)	3.37 ± 0.71	3.25 ± 0.55	0.408	3.92 ± 0.80	3.77 ± 0.48^**#**^	0.365
p53 (pg/mL)	92.40 ± 37.70	129.00 ± 43.50	**0.0003**	139.00 ± 52.70	177.00 ± 43.10^**#**^	**0.001**
p21 (ng/mL)	2.84 ± 0.60	3.15 ± 0.57	0.35	3.67 ± 2.00	5.27 ± 2.30^**#**^	**4.9** × **10**^**−5**^

^#^Significant on comparing khat chewers between groups (breast cancer vs. control) for telomerase (*P* = 0.001), p53 (*P* = 1.8 × 10^−5^), p21 (*P* = 2.2 × 10^−8^).

Significant values are given in bold.

The levels of telomerase, p53, and p21 in all the subgroups studied in both the control group and BC patients as compared to their corresponding non-OC user, non-khat chewer group are shown in Table 4. In the control group, telomerase in both OC users and those OC users + khat chewers were significantly (*P* = 2.8 × 10^−7^, *P* = 0.0002) higher by 33.7% and 23.6% as compared with the respective non-OC users non-khat chewers; whereas it was non-significantly different in the khat chewer of the control group. In contrast, telomerase was non-significantly higher in both OC users and the khat chewers groups of the BC patients by 6% and 4.2%; and non-significantly lower in the OC users + khat chewers group by 6.3% as compared with the respective non-OC users non-khat chewers.

**Table 4:** Effect of oral contraceptives, khat chewing and their combined use in breast cancer patients and control group.

	Control				*P* value vs. no OCs & non-khat chewers		
	No OCs & non-khat chewers (n = 20)	OC users (n = 20)	Khat chewers (n = 20)	OC users & khat chewers (n = 20)	OC users	Khat chewers	OCs & khat chewers
Telomerase (ng/mL)	2.88 ± 0.49	3.85 ± 0.55	2.94 ± 0.53	3.56 ± 0.36	**2.8** × **10**^**−7**^	0.775	**0.0002**
p53 (pg/mL)	84.30 ± 25.10	100.00 ± 46.40	123.00 ± 47.90	136.00 ± 38.70	0.25	**0.006**	**0.0002**
p21 (ng/mL)	2.63 ± 0.65	3.04 ± 0.48	3.05 ± 0.36	3.24 ± 0.72	0.34	0.33	0.16

	Breast cancer patients				*P* value vs. no OCs & non-khat chewers		
	No OCs & non-khat chewers (n = 15)	OC users (n = 15)	Khat chewers (n = 15)	OC users & khat chewers (n = 15)	OC users	Khat chewers	OCs & khat chewers
Telomerase (ng/mL)	3.81 ± 0.73	4.04 ± 0.88	3.97 ± 0.52	3.57 ± 0.35	0.26	0.42	0.25
p53 (pg/mL)	125.00 ± 56.30	152.00 ± 46.90	166.00 ± 45.40	188.00 ± 39.00#	0.09	**0.012**	**0.0001**
p21 (ng/mL)	2.99 ± 1.05	4.35 ± 2.49	4.12 ± 1.37	6.41 ± 2.50#	**0.007**	**0.023**	**8.0** × **10**^**−6**^

^#^Significant on comparing khat chewers + contraceptive pill between groups for p53 (*P* = 0.001), p21 (*P* = 2.2 × 10^−10^).

Significant values are given in bold.

P53 in both khat chewers and those that were OC users and khat chewers of the control group were significantly (*P* = 0.006, *P* = 0.0002) higher by 45.9% and 61.3% as compared with the respective non-OC users non-khat chewers; whereas it was non-significantly higher in the OC user group by 18.6%. Similarly, p53 in khat chewers and those that were OC users and khat chewers of the BC patients were significantly (*P* = 0.012, *P* = 0.0001) higher by 32.8% and 50.4% as compared with the respective non-OC users non-khat chewers group; whereas it was borderline-significantly higher in the OC user group by 21.6% (Table 4).

The levels of p21 were non-significantly higher in all the tested subgroups of the control group by 15.6%, 16%, and 23.2% as compared with the respective non-OC users non-khat chewers group. In contrast, the three tested subgroups: OC users, khat chewers, and the combined OC users and khat chewers groups of the BC patients were significantly (*P* = 0.007, *P* = 0.023, *P* = 8.0 × 10^−6^) higher by 45.5%, 37.8% and 2.1-folds as compared with the respective non-OC users non-khat chewers group (Table 4). However, p53 and p21 were significantly (*P* = 0.001, *P* = 2.2 × 10^−10^) higher in the OC users + khat chewers of the BC patients than the corresponding subgroup of the control group by 38.2% and 97.8%, respectively.

Using logistic regression showed telomerase to be a risk factor (OR 4.4, 95% CI 2.1–9.4, *P* = 0.0001) for BC. Furthermore, the use of OCs was a high-risk factor for increasing telomerase (OR 27.8, *P* = 0.002); whereas khat chewing seemed to be protective (OR 0.142, *P* = 0.012). Moreover, the combined use of OCs and khat chewing decreased the risk factor of high telomerase from OR 27.8 to OR 2.1 (*P* = 0.015) (Table 5). Furthermore, khat chewing is associated with a higher likelihood of p53 expression than that of OC users (OR 5.2, *P* = 0.004 vs. OR 2.2, *P* = 0.22), whereas the combined use of OCs and khat chewing was no different from that of the OC user group.

**Table 5:** Risk factor for changes in telomerase levels in control group.

	OR	95% CI	*P* value
Oral contraceptive	27.8	3.20–240	**0.002**
Khat chewing	0.142	0.03–0.66	**0.012**
Oral contraceptive + khat chewing	2.1	1.2–4.0	**0.015**

Significant values are given in bold.

*OR* odds ratio, *95% CI* confident interval.

## Discussion

The results presented in our study show BC patients to have raised levels of telomerase, p53, and p21. The significantly higher telomerase activity in our BC patients is in agreement with earlier studies reporting relative telomerase activity in malignant breast tumors when compared with benign ones^51–53^. Telomerase activity has been reported to be present in 59–93% of breast carcinomas, while it is not expressed in most normal cells^39,43,51^. A systematic review also showed that telomerase activity was significantly present in BC when compared with normal breast tissue or benign breast lesions^54^. This increase in telomerase activity could be attributed to its role in maintaining the telomere length which is required for the continuous growth of the tumor, especially in advanced tumors. Long telomeres may allow damaged cells to survive longer and continue to divide resulting in malignant transformation^55^. Several studies showed that longer leukocyte telomere length was positively associated with an increased risk of BC^56–60^. Accordingly, telomerase activity was suggested to be a potential diagnostic^39,61,62^ and prognostic marker of invasive ductal breast carcinomas^40,44,63^. Besides, telomerase was reported to correlate with tumor size and axillary lymph node status, and tumor stage^51,52,64–66^.

Moreover, both serum p53 and p21 were significantly raised in our BC patients. The significantly higher serum p53 level in BC patients is in line with previous studies reporting the presence of p53 in 30–69% of cases of ductal carcinoma of the breast, with a significant correlation between the p53 overexpression and histological grades of BC as well as with Bcl-2^67–71^. This increase in p53 may be attributed to cellular stress and DNA damage in BC patients. Acting as a first-line tumor suppressor, p53 initiates cell cycle arrest and apoptosis in response to DNA damage and oxidative stress^48,72,73^. The p53 protein induces apoptosis and exerts its growth inhibitory function via transactivation of particular cell cycle regulatory genes, such as p21, which leads to tumor growth arrest through inhibition of cyclin-kinase complex^49^. Consequently, the observed significantly higher serum p21 in our BC patients supports the findings that p21 expression may be p53-dependent and that it was up-regulated by p53 following DNA damage^68,74,75^. p21 is a downstream target of the p53 protein and the p53–p21 axis is an established central pathway for cell cycle inhibition and tumor suppression^50^.

On examining the effect of OC use on the tested parameters in both the control group and BC patients; telomerase was significantly higher in the control group who are on OCs, which is in agreement with earlier studies reporting sex hormones (androgen and estrogen) to be directly involved in telomerase activation by modulating the expression of genes involved in telomere maintenance, such as human telomerase reverse transcriptase (hTERT) and telomerase RNA component (TERC) as well as the post-transcriptional modification by serine/threonine protein kinase-dependent phosphorylation of hTERT^76–78^. The finding that telomere length is longer in nulliparous women raised the question of whether telomerase is activated in these women and whether this activation is mediated by prolonged estrogen signaling^79^. Estrogen deficiency^80^ and shorter duration of reproductive years^81^ have been associated with shorter leukocyte telomere length in peripheral blood. Moreover, estrogens and progesterone play a key role in promoting the proliferation of breast tissue during normal breast development, pregnancy, and breast carcinogenesis^82^. Three major mechanisms are hypothesized to be involved in their carcinogenic effects: stimulation of cellular proliferation via their receptor-mediated hormonal activity, direct genotoxic effects by increasing mutation rates through a cytochrome P450-mediated metabolic activation, and induction of aneuploidy^82^. Activation of telomerase is an early event in the development of BC that may lead to cellular immortality, a critical and rate-limiting step in oncogenesis^83^.

In contrast, telomerase levels were not affected in the OC users of the BC patients; which may be in line with a study reporting telomerase to be slightly decreased in the estrogen/progesterone-treated immortalized human mammary epithelial cells (76N TERT cells)^84^. Thus, highlighting the moderate growth inhibitory effects of estrogen and progesterone on cancer cell growth in the 76N TERT cells. Other studies, however, suggested that OCs may increase the telomerase activity in BC cells and human endometrial cancer cells, which could promote their growth and survival^85,86^. Estrogen was reported to induce telomerase activity and hTERT mRNA expression through a Mitogen-activated protein kinase (MAPK) dependent pathway in endometrial cancer cell lines^85^. Moreover, an earlier study found that progesterone exerts a biphasic effect on hTERT expression, depending on the duration of exposure: hTERT mRNA is induced within 3 h but decreases after 12 h^86^. Exposure to progesterone for > 48 h antagonizes estrogen-induced activation of hTERT expression via the same mitogen-activated protein kinase-signaling pathway in breast and endometrial cancer cell lines expressing progesterone receptors^86^.

The discrepancy with our results might be attributed to either the combined estrogen and progesterone in the contraceptive pills used by our patients or the raised p53 in the BC patients using the OCs. Thus, supports the majority of the evidence, which suggests the involvement of p53 in the regulation of telomerase activity in mammary epithelial cells and BC^45–47^; although a few reports suggest that telomerase activity appears to be independent of p53 expression^87^. The molecular mechanisms of regulation of telomerase activity by p53 may involve the down-regulation of hTERT transcription or the interaction of p53 with Sp1 or other transcription factors^46^.

Unlike telomerase, p53 was non-significantly increased in the control group using OCs but significantly increased in BC patients using OCs. Our results are in agreement with other studies showing estrogen and progesterone to induce a persistent increase in p53-dependent apoptosis and suppress mammary tumors in BALB/c-*Trp53*+/− mice^88^ as well as in 76N TERT cells^84^. Strong p53 staining in mammary tissues of pre-irradiated estrogen and progesterone-treated mice was demonstrated^88^. However, the significant increase of p53 in the OC users of the BC patients could be a response to the damage produced by the cancer manifestation^89^ whereby p53-mediated responses to DNA damage in pre-γ-radiated mice increased dramatically following treatment with estrogen and progesterone through the TGF-β-dependent pathways. Moreover, progesterone has been reported to significantly inhibit cell proliferation and induce apoptosis in two ovarian carcinoma cell lines and markedly up-regulated p53 expression indicating involvement of p53 in progesterone-induced apoptosis^90^.

Like p53, p21 was non-significantly higher in the control group using OCs and significantly higher in BC patients using the OCs. This is in agreement with earlier findings reporting both p53 and p21 protein expression to be induced following the estrogen/progesterone and retinoid treatment in immortalized human mammary epithelial cells^84^ and in both rats and mice upon carcinogen challenge^91^. The p21 gene expression is usually induced and tightly controlled by the tumor suppressor protein p53^50,68,75^. Moreover, radiation-induced expression of p21 was increased progressively by treatment with estrogen and progesterone^89^, and radiation-induced p21 was also undetectable in BALB/c-Trp53^−/−^ mice treated with estrogen and progesterone confirming that p21 expression was p53-dependent.

On the other hand, khat non-significantly reduced the telomerase level in both the control group and BC patients, which may be attributed to its reported apoptotic effect^30,31^ suggesting that khat induces caspase-dependent apoptotic cell death, mitochondria damage, reduced proliferation, and morphological features of autophagy in various leukemic cells^32^. Khat was also reported to inhibit cell proliferation in normal human keratinocytes and fibroblasts preceded by oxidative stress characterized by a rapid increase in intracellular ROS and depletion of GSH^33^, as well as having an inhibitory growth effect which is dependent on the concentration of khat, and the duration of exposure to khat. Furthermore, Khat extract induced protein phosphorylation of signal transducers p38 *MAPK* and p53, followed by reduced cell proliferation and cell death^31^. p38 is activated upstream of p53 and may be pivotal in khat-induced abnormal differentiation of in vitro-reconstructed human normal buccal mucosa^92^.

Our results also show khat chewing to significantly increase p53 in both the control group and BC patients, which is in line with those results reporting khat to induce the tumor suppressor and cellular stress sensor p53 expression in normal human oral keratinocytes and oral fibroblasts^34^ and human ovarian adenocarcinoma SKOV3 cell line^35^ and facilitate apoptosis by a p53-dependent manner^34^. p53 accumulation was also demonstrated in leukocytes following khat-extract treatment^31^, whereas cathinone and its derivatives generally reduced total p53 levels, p53 Ser15 phosphorylation. Experiments using p53 knock-down and knock-out cells confirmed that p53 was mostly redundant in khat-mediated cell death in vitro. Khat was reported to induce phosphorylation and acetylation of p53 in both khat-treated acute myeloid leukemia cell lines sensitive (MOLM-13) or resistant (MV4-11) to khat-induced apoptosis, but an accumulation of the p53 full-length isoform was only seen in MOLM-13^93^. Khat-induced apoptosis may involve the induction of reactive oxygen species^33^, which is described to function upstream of p53 and p38 in some model systems. The generation of intracellular ROS by Khat induces the sustainable activation of JNK, which subsequently decreases cell viability and increases cell apoptosis^36^.

Moreover, p53 is highly sensitive to low levels of DNA damage and is therefore also upregulated in response to genotoxic stress^94^. Khat has been reported to be genotoxic in both human and animal studies^95^, as well as inhibiting cell growth. This genotoxic stress damage leads to the activation of kinases such as CHK1, CHK2, ATM, or ATR which in turn leads to phosphorylation of p53^96^. An earlier study reported that the cell cycle arrest observed by khat could be linked to increased p53 and p16 in response to its oncogenic and genotoxic stress^34^. It has been suggested that p53 and p21 mediate cell cycle arrest and senescence primarily in response to DNA damage, whereas p16 mediates senescence in response to oncogenic and mitogenic stress^97^. In normal unstressed cells, p53 is continuously synthesized, and its levels are kept low by ubiquitin-dependent degradation^72^. On exposure to cellular stress, p53 is stabilized and its transcriptional activity is induced. The p53 protein then activates signaling pathways involved in cell cycle arrest, DNA repair, or apoptosis^72^, hence preventing the propagation of defective genes or mutations that could have the potential for tumorigenesis.

In contrast, p21 was non-significantly increased in the control group and significantly raised in BC patients. Our results differ from earlier studies reporting a p21 increase with p53 in normal human oral keratinocytes and oral fibroblasts^34^. Moreover, an in vitro study using reconstructed human oral mucosa showed an early abnormal expression of p21 within the khat-treated samples when compared with controls that weakly expressed p21 suprabasally only, confirming the premature differentiation suggested by the histological changes^92^. p21 is thought to play a role in the induction of an irreversible cell cycle arrest and terminal differentiation, and normally it is expressed above the basal compartment only in oral squamous epithelium^98^. However, our results of the BC patients are in agreement with those reported in breast, prostate, cervical, and squamous cell carcinomas whereby p21 increased with p53^50,68^.

The effect of the combined use of OCs and khat chewing showed telomerase and p53 to be significantly increased in the control group, whereas p53 and p21 were significantly increased in BC patients. In the control group, the increased telomerase may largely be attributed to the effect of estrogen and progesterone in the contraceptive pills^51,87^; whereas the increase in p53 may be a response to the genotoxic stress of khat^94,96^. In BC patients, the increased p53 and p21 could be due to the additive effect of both the use of OCs and khat chewing.

Using logistic regression showed telomerase to be a risk factor (OR 4.4) for BC, and that the use of the OCs was a high-risk factor for increasing telomerase (OR 27.8). This is in agreement with many studies showing women taking OCs have a higher risk of BC than women who have never used the contraceptive pill^5,17^. In contrast, our results showed khat chewing to be protective (OR 0.142), and the combined use of OCs and khat chewing decreased the risk factor of high telomerase from OR 27.8 to 2.1. This protective effect was further highlighted by the higher likelihood of p53 expression with khat chewing, which may lead to induce a defense mechanism against DNA damage to improve repairing or via cell cycle arrest^31,34^.

In general, due to the adverse events of conventional therapeutic strategies, the identification of new therapeutic targets and anticancer agents is imperative. Natural products and their derivatives have emerged as promising sources for the development of new drugs due to their wide availability, diverse biological activities, and low toxicity and side effects^99^. Natural products and their derivatives applied to anti-cancer treatment have demonstrated reduced BC-related mortality and improved outcomes by modulating various cellular processes including the inhibition of DNA synthesis, angiogenesis, induction of cell apoptosis, increase in chemotherapy sensitivity, regulation of immune function, and reversal of drug resistance^100^. Nonetheless, sustained research efforts are still required to overcome their limitations for full therapeutic potential as the clinical application of natural products in treating BC is hampered by challenges such as low bioavailability and stability.

## Conclusion

Breast cancer is associated with a high level of telomerase and the use of OCs is a risk factor for increasing telomerase in normal subjects, whereas the combined use of OCs and khat chewing decreased the risk factor of high telomerase. The protective effect of khat chewing decreases the risk of high telomerase probably through increasing p53 expression suggesting that the stress sensor protein p53 and inhibitors of cyclin-dependent pathways could play a role in cell cycle arrest. Khat chewing appears to pose general stress, which could affect cell cycle regulation, differentiation, and even cell death by increasing the expression of p53 and p21, and the increase in p21 could be p53-dependent. While the study provides valuable insights including increased awareness of lifestyle factors affecting cancer risk, further research is needed to explore the mechanism underlying these effects.

## Data Availability

The data set generated and analyzed during this study is included in this submitted manuscript and is available from the corresponding author on reasonable request.
